# Extracellular vesicle-mediated transfer of miRNA-1 from primary tumors represses the growth of distant metastases

**DOI:** 10.1038/s12276-024-01181-7

**Published:** 2024-03-27

**Authors:** Chae-Yi Kim, Kang-Hoon Lee, Keun Hong Son, Tae-Jin Shin, Je-Yoel Cho

**Affiliations:** 1 Department of Biochemistry, College of Veterinary Medicine, Research Institute for Veterinary Science, and BK21 FOUR Future Veterinary Medicine Leading Education and Research Center, Seoul National University, Seoul, 08826 Republic of Korea; 2 Comparative Medicine Disease Research Center (CDRC), Science Research Center (SRC), Seoul National University, Seoul, 08826 Republic of Korea

**Keywords:** Breast cancer, Metastasis

## Abstract

Metastases originate from primary tumors and reach distant organs. Growing evidence suggests that metastases are under the control of primary tumors even outside the primary site; however, the mechanisms by which primary tumors remotely control metastases remain unclear. Here, we discovered a molecular mechanism by which primary tumors suppress metastatic growth. Interestingly, we found that extracellular vesicles (EVs) derived from the primary tumor can inhibit the growth of metastases both in vitro and in vivo. miR-1 was particularly enriched in primary tumor-derived EVs (pTDEs) and was found to be responsible for the suppression of metastatic growth. Mechanistically, intracellular reactive oxygen species (ROS) production and DNA damage were induced, which led to cell cycle arrest. Collectively, our data demonstrate that primary tumors restrict the growth of distant metastases via miR-1 in pTDEs and that miR-1 could potentially be used as an antimetastatic agent.

## Experimental and molecular medicine

Harnessing miR-1: A Potential Anticancer Drug to Combat Metastasis Cancer metastasis, or the movement of cancer cells from the initial tumor to other body parts, is a complicated process that we don’t fully comprehend. This research aimed to explore how the original tumors manage the growth of metastatic, or spreading, cancer cells. The scientists discovered that extracellular vesicles (EVs - tiny particles released by cells) produced by cancer stem cells in the original tumors can slow the growth of spreading cells. Specifically, these EVs carry a molecule named miR-1, which can cause DNA damage and cell cycle arrest in spreading cells, reducing their multiplication. The study also found that the amount of miR-1 in EVs increases with the advancement of breast cancer, suggesting its potential use for diagnosis and treatment. This study offers new understanding into how cancer spreads and suggests new possibilities for cancer treatment. This summary was initially drafted using artificial intelligence, then revised and fact-checked by the author.

## Introduction

Metastases that have migrated from primary tumors have three potential fates after settling in a secondary organ. First, they may undergo metastatic outgrowth; second, they may die via mechanisms involving immune cells or apoptosis; and finally, they may take on a dormant state; metastasis accounts for approximately 70% of cancer-related deaths^1,2^. Metastases that undergo outgrowth can be effectively targeted with antiproliferative drugs. However, targeting dormant cancer cells is a challenge, as clear treatment targets have not been defined^3^. Even after metastatic cells have migrated from the primary tumor and settled elsewhere, they remain under the control of the parental primary tumor. Primary tumors play dual roles: they promote the dissemination of metastatic cells from the primary site while also maintaining control of their behaviors^4–6^. Many studies have revealed how primary tumors influence the tumor microenvironment, thereby restraining metastatic growth through various dormant mechanisms, such as interfering with angiogenesis (angiogenic dormancy) or eliminating by immune cells (immunologic dormancy)^7–9^. One fundamental finding is that angiostatin and endostatin interrupt metastatic angiogenesis and lead to nutrient starvation^10,11^. Furthermore, primary tumors indirectly restrict metastasis by remodeling CD8+ cytotoxic T cells and macrophages to eliminate them^4,6,12^. Consequently, one pivotal factor contributing to the sudden increase in metastases after primary tumor treatment may be the lack of metastatic growth inhibition signals from the primary tumor.

Since the discovery of extracellular vesicles (EVs), cells have been known to deliver their signals to distance cells by wrapping such signals in a lipid bilayer^13^. EVs contain proteins, lipids, and nucleic acids. Research on the biological function of EVs in cancer has focused mainly on their role in tumorigenesis and the formation of the tumor microenvironment^14^, and there have been a few attempts to address the function of EVs as controllers of metastases^15–18^. Despite numerous studies on tumor-derived EVs (TDEs), there is no evidence showing the role of the primary TDEs (pTDEs) during metastasis.

In this study, we demonstrated that primary tumors use EVs to control metastatic growth. When small RNA sequencing was performed, miR-1 was found to be more abundant in pTDEs than in metastatic tumor-derived EVs (mTDEs). Additionally, we observed a reduction in the expression of miR-1 target genes in metastatic cancer cells treated with pTDEs. Moreover, we found that exosomal miR-1 acts as a key regulator of tumor inhibition and that engineered pTDEs with high levels of miR-1 markedly inhibited metastasis in both cell and mouse models. These findings highlight the role of miR-1 in pTDEs in metastastic growth inhibition.

## Materials and methods

### Cell culture

Six canine mammary gland adenocarcinoma cell lines (CHMp, CHMm, CIPp, CIPm, CTBp, and CTBm) were purchased from the N. Sasaki laboratory^19^ and grown in RPMI 1640 medium (HyClone, SH30027) supplemented with 10% fetal bovine serum (FBS; Gibco 1600044) and 50 μg/ml gentamicin (Sigma‒Aldrich, G1272). CHMp and CHMm cells were transfected with a firefly luciferase gene-expressing plasmid (Addgene #18964) using Lipofectamine 3000 reagent (Invitrogen, LM3000015). Culture medium containing geneticin (G418 sulfate) was used to select stably transfected cells (Gibco, 10131035). Twenty-four hours after transfection, the cells were supplemented with G418 (500 µg/ml) and maintained for one week to eliminate untransfected cells. Single cells were isolated from stable luciferase-expressing cells to establish a stable cell line and maintained with 250 µg/ml G418 for two weeks. Luciferase expression was confirmed with a luciferase assay. Human umbilical vein endothelial cells (HUVECs) were grown in endothelial cell basal medium-2 (EGM-2, Lonza, CC-3156) supplemented with EGM™-2 singleQuots® (Lonza, CC-4176). All cells were grown in a humidified incubator at 37 °C with 5% CO_2_ and confirmed to be negative for mycoplasma contamination.

### Mouse experiments

All mouse experiments were conducted in accordance with the Seoul National University Institutional Animal Care and Use Committee (IACUC) guidelines, and the animal protocol was approved (SNU-210323-1-2). Nude mice (CrTac:NCr-Foxn1nu) were kept in pathogen-free conditions and maintained under a 12 h light/dark cycle at a controlled room temperature (22 ± 2 °C). A metastasis model was generated in 5-week-old female nude mice following orthotopic injection of a total of 5 × 10^5^ luciferase-labeled CHMp and CHMm cells into the mammary fat pad, and resection surgery was performed 21 days postimplantation. Mice were anesthetized, and primary tumors were resected. Mice that underwent surgery were monitored for symptoms of pain and were sacrificed via inhalation of carbon dioxide (CO_2_). IVIS bioluminescence imaging was carried out during the formation of spontaneous lung metastases. All mice were randomized before injection of EVs and blindly selected before injection. For EV injection, all the treatments were administered via intravenous injection in a final volume of 150 µl. Mice were treated with EVs six times at two-day intervals. The experimental endpoint was established according to IACUC guidelines, and the maximal tumor volume was never exceeded.

### Extracellular vesicle isolation and labeling

EVs were purified from cells cultured under serum-free conditions using a combination of ultrafiltration and ultracentrifugation. The graphical method is illustrated in Supplementary Fig. 2a. The cells were grown to 80% confluence, washed two times with PBS and incubated in serum-free medium for 24 h. First, the cell culture supernatant was subjected to differential centrifugation to eliminate cells, dead cells, and cell debris and filtered sequentially with 0.45 and 0.22 μm filters. The filtered supernatant was concentrated using 10 K Amicon Ultra 15 Centrifugal Filter Units (Merck, UFC903024). The filtered units were sequentially centrifuged. The mixture was ultracentrifuged for 80 min and washed with PBS. An EV concentration of 0.1 µg/ml was used in all in vitro assays.

EVs were isolated from biological fluids using the following commercial kit: ExoQuick exosome precipitation solution (System Biosciences, SBI-EXOQ5A-1). The plasma and serum samples were centrifuged at 3000 × *g* for 15 min to remove cells and cell debris. ExoQuick was added to the supernatant at an appropriate volume and incubated for 30 min at 4 °C. Pelleted EVs were resuspended in Qiazol (Qiagen, 79306) for RNA isolation and in urea/SDS lysis buffer for protein isolation. The isolated EVs were stored at −80 °C for later use.

EVs were labeled with PKH67 lipophilic membrane dye (Sigma, MNI67-KIT) following the manufacturer’s instructions. In brief, isolated EVs were resuspended in 1 ml of Diluent C, after which 6 µl of PKH67 dye was added. The mixture was incubated for 5 min at room temperature and centrifuged at 100,000 × *g* for 80 min.

### Nanoparticle tracking analysis (NTA)

NTA was used to characterize the size and concentration of EVs from the cell culture supernatant and biological fluids using the NanoSight LM10 model (Malvern). The samples were diluted with PBS (0.22 µm filtered) and injected into the laser chamber. The data were analyzed by NTA v3.2 software.

### Transmission electron microscopy (TEM)

The morphology of the EVs was analyzed by TEM using a Talos L120C system (Czech). Briefly, the samples were stained with a negative staining method using 2% uranyl acetate. One drop of diluted EVs was dropped on a glow-discharged copper/carbon-coated grid. After 1 min, the grid was drained using filter paper, and one drop of 2% uranyl acetate was added. The staining solution was removed, and the samples were observed via TEM (120 kV).

### Small RNA sequencing and data analysis

The miRNA-seq library was prepared using the Small RNA Library Prep Kit (Nextflex) and sequenced as 100 bp or 150 bp paired-end reads on the Illumina HiSeq 3,000 and NovaSeq 6,000 platforms. To remove adapters with low-quality reads and extract miRNA-specific sequences, cutadapt was used with the following options: quality-base 33-u 4-m 22-M 30-f fastq-q 20-O 6-j 23-a adapter sequence. In this step, two different adapter sequences (TGGAATTCTCGGGTGC-CAAGG and GATCGTCGGACTGTAGAACTCTGAAC) were used for forward and reverse paired-end sequencing. Because trimmed reads are short (22–30 bp), forward and reverse reads in the same sample were merged into one fastq-formatted file. Before and after the trimming step, the quality of the sequenced reads was estimated using FastQC.

For known and novel miRNA analysis, the miRDeep2 package was used. Before analysis, the following two necessary files were prepared: (1) sequence files of mature and hairpin forms of dog miRNAs, which were downloaded from the miRbase database and extracted using the extract_miRNAs.pl script; and (2) indexed files from the dog reference genome (CanFam3.1) using Bowtie. First, all filtered read data were merged into one file for novel miRNA analysis. The merged data were converted to a collapsed FASTA-formatted file and aligned to the reference genome using the mapper.pl script with the options (-e -h -j -m -p). Second, novel miRNAs were identified using the miRDeep2.pl script. The identified mature and hairpin forms of novel miRNAs were extracted and combined with known forms of miRBase prepared previously. Finally, the expression values of known and novel miRNAs were calculated using the quantifier.pl script. The counts per million (CPM) values, which are scaled by the total number of reads, were used for further analysis. MiRDeep2 analysis provides a miRNA score ranging from -10 to 10; a higher score represents a genuine miRNA. We set a cutoff of 4 for strict identification of novel miRNAs. To estimate the reproducibility of the data between replicate samples, Pearson correlation values were calculated and visualized using the correlation function in R. For differentially expressed miRNA analysis, fold changes in expression and significance (P value) were calculated using the EdgeR package in R. With these calculated values, a volcano plot was generated through the ggplot package in R.

### miR-1 mimic transfection

Synthetic microRNA mimics were used to overexpress and overload microRNAs in cells and EVs. CHMm cells were transfected using the lipid carrier Lipofectamine RNAiMAX (Invitrogen, 13778150) following the manufacturer’s instructions. Fifty picomoles of miR-1 mimics were mixed with RNAiMAX reagent and then incubated for 15 min at RT. The miR-1 and RNAiMAX complexes were added to the cells, which were then incubated for 24 h at 37 °C in a CO_2_ incubator. EVs were transfected using 0.3 M CaCl_2_ following the modified CaCl_2_-mediated transfection method^20^. Forty micrograms of EVs was mixed with 100 pmole of miR-1 mimics in BPS supplemented with 0.3 M CaCl_2_ and incubated on ice for 30 min. Then, the mixture was heat-shocked at 42 °C for 60 s and incubated on ice for 5 min. Transfected EVs were isolated again by ultracentrifugation and washed with PBS.

### Clinical specimens

All study protocols and specimen collection steps were approved by the Institutional Review Board (IRB). The IRB was approved by the Seoul National University (IRB#SNU 16-10-063). Blood sample collections from 9 healthy controls and 31 breast cancer patients performed in accordance with established guidelines. Informed consent for specimen collection was obtained from all subjects, including both humans and dog guardians, when they were enrolled.

### Statistical analysis

The data represent the mean ± SEM. Statistical analysis was performed using Prism software (v.8.0.1, GraphPad Software). To assess the statistical significance of differences among multiple groups, 2-way ANOVA combined with Tukey’s honest significant difference (HSD) test was performed, and Student’s *t* test was performed for comparisons between CHMp and CHMm cells. Significant differences are indicated with different symbols (*P < 0.05, **P < 0.01, and ***P < 0.001) in each figure legend. The number of experimental repeats and the value of n are also indicated in the figure legends.

## Results

### EVs from primary tumors inhibit the growth of metastases

To investigate the role of pTDEs in controlling metastases, we used two types of metastatic models. The first model is the spontaneous recurrence model, in which metastases grow after surgical removal of the primary tumor. For the second model, cell lines of both primary and metastatic tumors established from the same spontaneous cancer patient were used. Since there is no pair of naturally occurring human breast cancer (HBC)-derived primary and metastatic tumor cell lines, we used cells derived from a canine mammary gland tumor (CMT), as this tumor type has recently been reported to have pathological and molecular aspects similar to those of HBC^21,22^. The pair of CMT cell lines (primary/metastatic, CHMp/CHMm) originated from the same patients. In contrast to previous studies on primary tumors and metastases, we used cell lines derived from the primary tumor and metastasis of a single subject to increase the accuracy of our analysis.

**Fig. 1:**
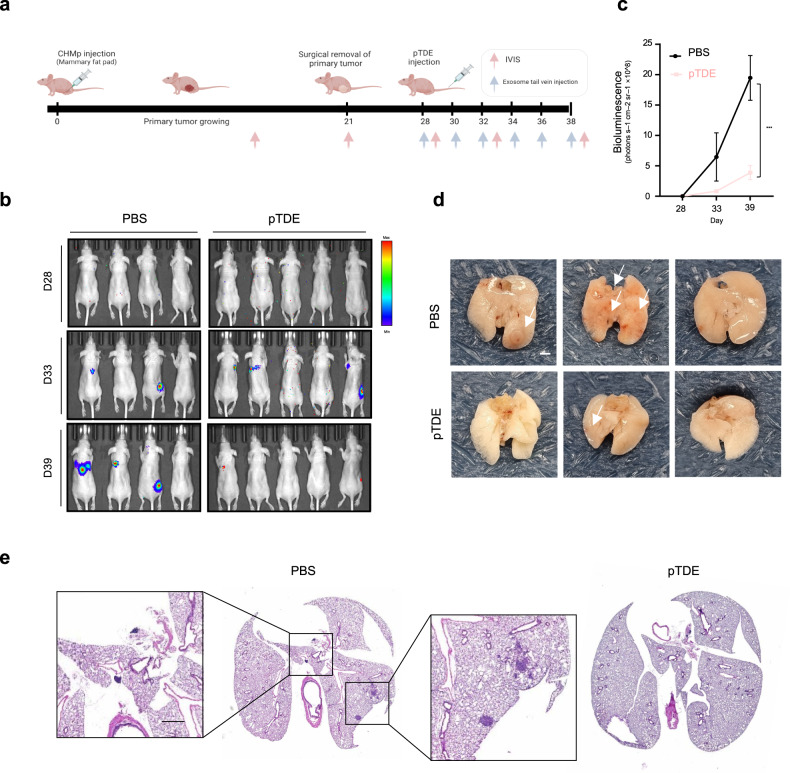
Primary tumor-derived EVs (pTDEs) restrict metastasis formation and growth. **a** Schematic illustration of the established experimental metastasis model. The mice were inoculated with CHMp cells into the mammary fat pad to produce a primary tumor on Day 0 (*n* = 20). On Day 21, the primary tumors were surgically removed. Seven days after surgery, PBS (*n* = 4) or pTDEs (*n* = 5) were administered to the mice. Mice were treated with PBS or pTDEs (10 µg/mouse) through the tail vein six times every two days. **b** Bioluminescence images of CHMp lung metastases after surgery for confirmation of residual primary tumor mass (at D28). D33 bioluminescence images were acquired from the mice treated with PBS or pTDEs three times, and D39 images were acquired six times at two-day intervals. For each group of daily images, four mouse images were acquired at four positions (dorsal, ventral, right lateral and left lateral) to capture every possible signal from the mice. **c** Graph showing the quantification of lung metastases in a mouse model of metastasis treated with PBS and pTDEs. Unpaired Student’s *t* test was used to compare groups. (***P < 0.001) **d** Representative images of lungs after PBS and pTDE treatment. Top: PBS-treated samples; bottom: pTDE-treated samples. Scale bar, 2 mm. **e** Representative H&E staining of lung tissue (4X) from the metastasis model. The left panel shows the PBS-treated mice, and the right panel shows the pTDE-treated mice. Magnified images: Scale bar, 1 mm. The results are presented as the mean ± SD (4–5 mice were used for each group). Figure 1a was created with BioRender.com.

First, for the metastatic mouse model, we used surgical methods described by Piranlioglu et al.^6^. Primary tumors were surgically excised 21 days after CHMp cell injection (Supplementary Fig. 1a–c). After surgery, mice that underwent sham surgery exhibited cancer growth concentrated at the primary site rather than developing distant metastases (Supplementary Fig. 1d). In contrast, the majority of the mice were confirmed to be free of residual primary tumors and were injected with pTDEs or the control. pTDEs were isolated as described in the Materials and Methods section, and the purified EVs were cup-shaped and positive for TSG101 and Alix (Supplementary Fig. 2a–d). PKH67-labeled pTDEs were found to be highly enriched in disseminated tumors and primary tumors (Supplementary Fig. 3a). The injection schedule is depicted in Fig. 1a. Subsequently, the mice were divided into two groups: one group was regularly injected with pTDEs, and the other group was injected with PBS as a control. Bioluminescence imaging revealed little metastatic growth in the pTDE group compared to the control group on the 39th day (Fig. 1b, c; Supplementary Fig. 3b). Metastases occurred in 3 of the 4 control mice, but very minor signals were observed in 2 of the 5 mice injected with pTDEs (Fig. 1b, c). There were more metastatic nodules in the lungs of the control mice than in those of the pTDE-injected mice (Fig. 1d, e). In a mouse model, the removal of primary tumors was shown to promote the growth of metastatic cancers in the clinic^23–26^. By comparison with the negative control, we demonstrated that when pTDEs were injected into spontaneous metastases generated after the removal of the primary tumor, they exerted an inhibitory effect on tumor growth, even in metastases that were previously undetectable. Collectively, our data reveal the pivotal role of pTDEs in metastatic growth inhibition.

### pTDEs autonomously suppress metastatic growth in direct and indirect manners

**Fig. 2:**
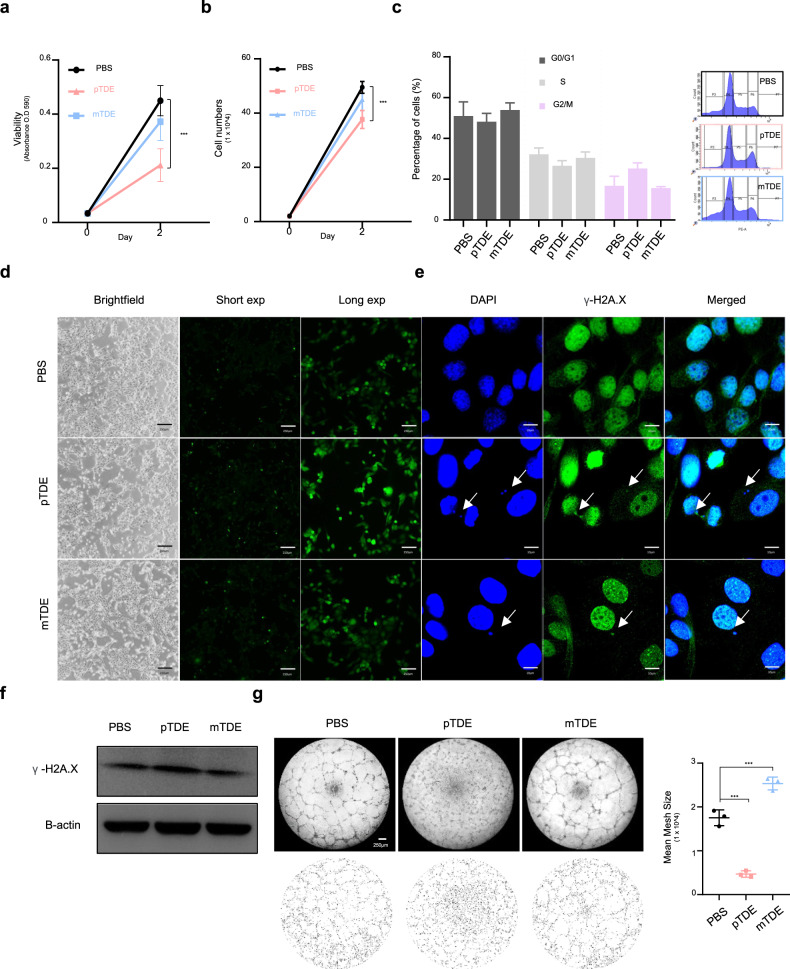
pTDEs induce cellular ROS generation, DNA damage, and G2/M arrest, resulting in the inhibition of proliferation in recipient cells. **a** Recipient cell viability after tumor-derived EV (TDE) treatment (0.1 µg/ml) was measured by MTT assays (*n* = 8). **b** The proliferation rate was manually measured by cell counting in the PBS and TDE treatment groups. pTDEs decreased cell viability and proliferation. **c** Cell cycle analysis of exosome-treated cells was performed using flow cytometry. pTDEs induced an increase in the proportion of cells in G2/M phase. **d** Representative images showing cellular ROS levels. Intracellular oxidative stress was measured with the fluorescent ROS probe 2,7-dichlorodihydrofluorescein diacetate (H2DCFDA). A brighter green fluorescence indicates high ROS. EV-treated cells were stained with H2DCFDA to measure intracellular ROS. Scale bar, 170 μm. **e** For measurement of damaged DNA, γ-H2A.X (green) and DAPI (blue) were stained in the EV-treated CHMm cells. The white arrow indicates damaged DNA, which colocalized with γ-H2A.X and DAPI. Scale bar, 10 μm. **f** Western blot for intracellular γ-H2A.X, γ-H2A.X increased with pTDE treatment. **g** Tube formation assays of HUVECs to detect the angiogenic potential of pTDEs. HUVECs were seeded on Matrigel, and the exosomes were treated for 24 h. Representative images showing that pTDEs inhibited tube formation compared with that of the controls. Bottom, microscopy images were analyzed by the ImageJ plugin Angiogenesis Analyzer. The quantification of the mean mesh size is presented as the mean ± SEM. Two-way ANOVA and Tukey’s HSD test were used to compare groups; **P < 0.01, ***P < 0.001, and ns not significant. The results are presented as the mean ± SD.

Since primary tumors affect metastatic cancer cells both directly and indirectly through immune cells and endothelial cells that constitute the tumor mass, we next examined the impact of treating cancer cells and endothelial cells with pTDEs. We used a second metastatic model in which CHMm (metastatic) cancer cells were treated with pTDEs or mTDEs as a control to rule out any influences resulting from changes in the process of EV purification. CHMm cells treated with pTDEs exhibited reduced viability and proliferation, whereas the viability and proliferation rates of CHMm cells treated with mTDEs were not significantly different from those of the PBS control group (Fig. 2a, b). Moreover, more pTDEs accumulated in the G2/M phase of the cell cycle in the pTDE treatment group than in the control group (Fig. 2c). Since the accumulation of cells in the G2/M phase can be caused by cellular stress, such as cell oxidation, DNA replication, and transcription^27,28^, we investigated whether pTDE treatment induces cell stress. Treatment with pTDEs caused a marked increase in intracellular ROS levels (Fig. 2d) and in the level of γ-H2A.X, a sensitive marker of damaged DNA (Fig. 2e, f). These results indicate that pTDEs induce an increase in intracellular reactive oxygen species (ROS) levels, genomic instability, and accumulation in the G2/M phase and suppress the growth of metastatic cells. However, this growth inhibition effect was not related to cell death or migration (Supplementary Fig. 4a–c).

In contrast, pTDEs had a strong effect on the tube formation of endothelial cells (Fig. 2g). Human umbilical vein endothelial cells (HUVECs) incubated with pTDEs showed a 1.5-fold decrease in the area of meshes compared to that in the cells incubated with PBS, which formed regular tubes. These findings revealed that pTDEs inhibit metastasis through direct inhibition of metastatic tumor cell growth in mice and indirectly influence endothelial cells in the tumor microenvironment through the inhibition of angiogenesis.

### Primary tumor stem cell-derived EVs restrict metastatic growth

**Fig. 3:**
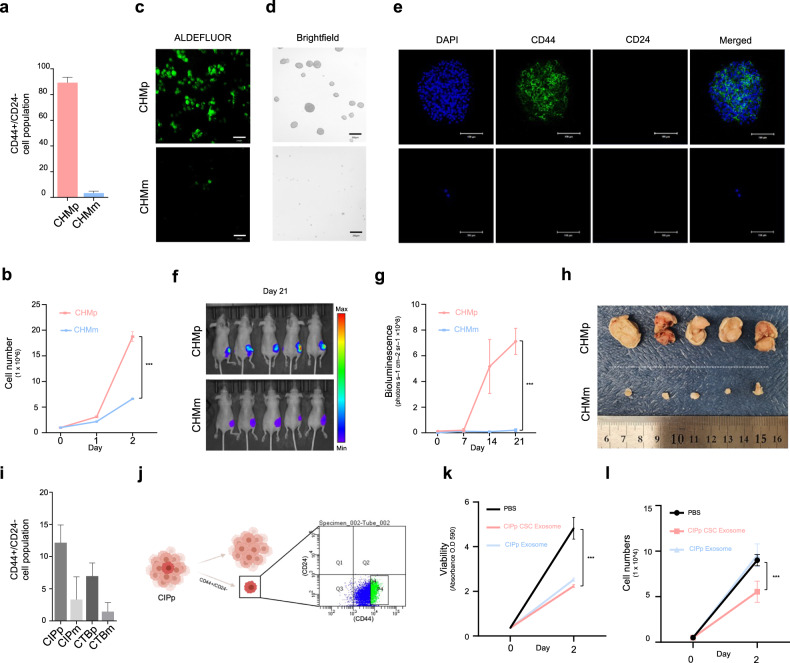
Cancer stemness contributes to the suppression of metastases. **a** CMT cell lines were stained with CD44 and CD24 antibodies and analyzed by flow cytometry. **b** Growth rates of the CHMp and CHMm cells. Cell proliferation was measured via manual cell counting. **c** ALDH activity was examined using an ALDEFLUOR assay in CHMp and CHMm cells. Fluorescence images were examined using a 40x objective lens; the scale bar indicates 130 μm. **d** Representative confocal microscopy images of mammospheres formed by CHMp and CHMm. Scale bar, 400 μm. **e** Mammospheres were stained with antibodies against the CSC markers CD44 (green) and CD24 (red). DAPI (blue) was used as a nuclear marker. Scale bar, 100 μm. **f** Whole-body IVIS bioluminescence images on Day 21. Mice were injected with equal numbers of CHMp (top) and CHMm (bottom) cells on the mammary fat pads (*n* = 5 per mouse group). **g** Graph showing the total flux of IVIS-treated mice used to measure their growth ability. The primary tumor volume was quantified once a week. **h** Picture of surgically removed CHMp (top) and CHMm (bottom) primary tumor masses. **i** Flow cytometry analysis of CMT cell lines with CD44 and CD24 antibodies. **j** Schematic illustration of the isolation of the CSC population (CD44+/CD24−) from the CIPp cell line. **k** An MTT assay was conducted to compare the viability of cells treated with CIPp exosomes and CIPp-CSC exosomes. **l** The proliferation rate of cells treated with PBS or exosomes was determined by manual cell counting. CIPp-CSC exosomes resulted in a greater decrease in the proliferation rate of CIPm cells than did CIPp exosomes. All the data are presented as the mean ± SEM. Experiments were performed in triplicate unless otherwise indicated. The statistical analysis is presented. **P < 0.01 and ***P < 0.001. ns not significant. The results are presented as the mean ± SD (5 mice were used for each group).

It is speculated that cells that disseminate from primary tumors and settle in distant locations are less likely to thrive in harsh environments if they lack stemness features^29–32^. Therefore, we examined the cancer stemness features of primary tumors to determine the inhibitory effect of these tumors on metastasis. Although many studies have investigated the effect of primary cancer stem cell-derived EVs on disseminated cancer cells (DTCs), no reports have clearly addressed the antitumor effects of these EVs. To further investigate how primary tumors control metastases, we compared the stemness features of primary and metastatic tumors (Supplementary Fig. 5a). The ${{CD}44}^{+}$/${{CD}24}^{-}$ expression pattern, which is representative of breast cancer stem cells (CSCs), was observed for a greater percentage of CSCs in the primary tumor than in the metastatic tissue (Fig. 3a). In addition, other breast CSC characteristics, such as rapid cell proliferation, high aldehyde dehydrogenase (ALDH) enzyme activity, and mammosphere formation, were substantially enhanced in CHMp cells vs. in CHMm cells (Fig. 3b–d). The spheroids were fluorescently labeled with CD44 and CD24, and only the CSC populations could form spheroids (Fig. 3e). We further compared the CD44, CD24, and ALDH1A1 protein levels and the levels of CSC-related genes between CHMp and CHMm cells (Supplementary Fig. 5b, c and Supplementary Table 1); the assessed genes included CD44, ALDH, drug resistance-related ABC transporters, stemness factors, and epithelial–mesenchymal markers. Interestingly, compared with CHMm cells, CHMp cells had higher CD44, ALDH, and ABCG2 expression levels and higher levels of mesenchymal markers. Moreover, the CSC-rich CHMp cells formed larger and faster-growing tumors than did the CHMm cells and showed vast differences even when the same number of cells were inoculated (Fig. 3f–h and Supplementary Fig. 5d). All these data suggest that primary tumor CHMp cells have more CSCs than CHMm cells.

We further investigated whether primary tumors with stemness features produced distinct EVs that could exert an antimetastatic growth effect. We further used other primary and metastatic cells, CIPp and CIPm cells (Supplementary Fig. 5a), which exhibited fewer differences in CSC features (Fig. 3i). Notably, there was no significant difference in the expression of genes associated with ALDH or drug resistance- or stemness-related transcription factors except Nanog between CIPp and CIPm cells (Supplementary Fig. 5e). Thus, we sorted the population of ${{CD}44}^{+}$/${{CD}24}^{-}$ cells, which we named CIPp-CSCs (Fig. 3j). EVs were isolated from the maintained CIPp-CSC portion and added to CIPm cells. CIPp-CSCs decreased cell viability (Fig. 3k) and inhibited proliferation (Fig. 3l). CIPp-CSC-derived EVs significantly suppressed metastatic cell proliferation compared with CIPp-derived EVs. These data confirmed that the growth-inhibitory effect of EVs derived from primary tumors is associated with the stemness of the CSCs that constitute the primary tumor. Therefore, we examined the cancer stemness features of primary tumors to determine the inhibitory effect of these tumors on metastasis. Analyses of cancer stemness features in the primary tumor cell lines CHMp and CIPp revealed that nearly 80% of CHMp cells present cancer stemness features (${{CD}44}^{+}$/${{CD}24}^{-}$/${{ALDH}}^{{high}}$); CIPp also has a greater proportion of cells presenting stemness features than its metastatic cell line, but the proportion is not as high. Moreover, EVs derived from the *CD*44^+^/*CD*24^−^ population of CIPp cells had markedly greater antitumor activity than EVs derived from the whole CIPp cell population, suggesting that this *CD*44^+^/*CD*24^−^ population is involved in the suppression of metastasis.

### miR-1 is enriched in pTDEs and suppresses target gene expression in recipient metastases

To determine which factors play a role in the effects of pTDEs, we performed small RNA sequencing (sRNA-seq). The procedures for RNA acquisition and QC data from small RNA sequencing are described in Supplementary Fig. 6a–e.

**Fig. 4:**
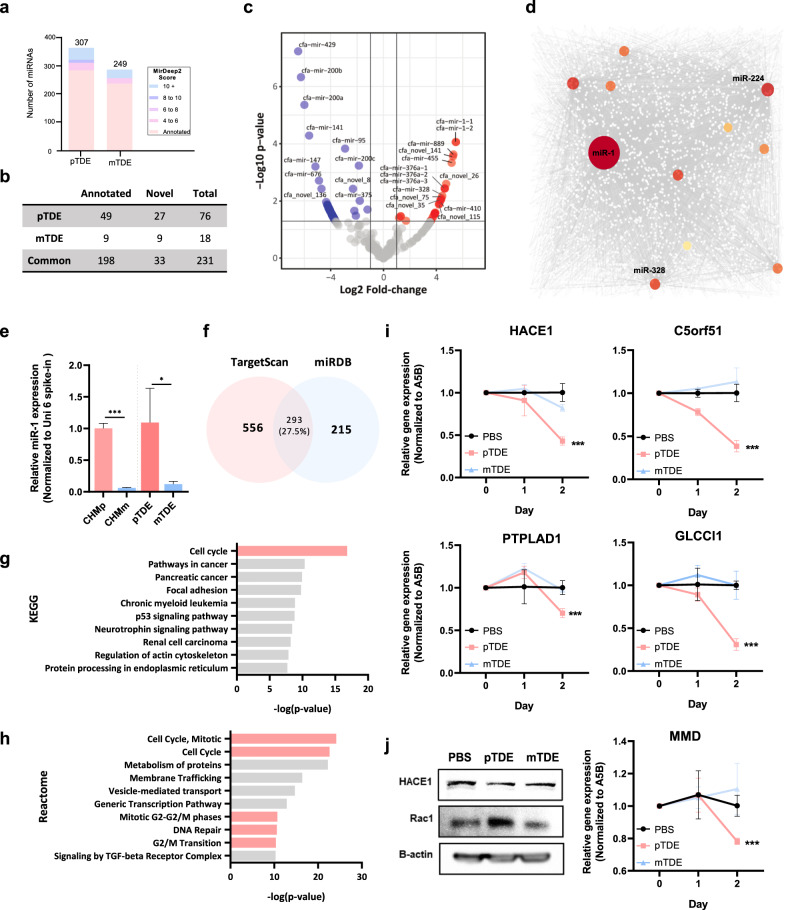
Small RNA sequencing revealed that miR-1 is enriched in pTDEs and that its network plays a critical role in cell cycle regulation. **a**, **b** Comparison of the identified miRNAs between pTDEs and mTDEs. **c** Volcano plot showing differentially expressed miRNAs between pTDEs and mTDEs. The red dots represent pTDE-enriched miRNAs, while the blue dots represent mTDE-enriched miRNAs. The gray dots represent miRNAs with -log10 (*p* value) and -log2 (fold change) values less than 1.3 and 1, respectively. **d** Network analysis of miRNAs and their target genes was conducted for the top ten pTDE miRNAs. The size of the colored dots indicates the number of genes regulated by the miRNA. miR-1 has the largest colored dots, which represent more interactions than others. **e** Comparison of miR-1 expression between CHMp and CHMm cells at the cellular level (left) and in exosomes (right). EVs were treated with RNase for 10 min at 37 °C to degrade contaminating EV-free miRNAs. Threshold cycle (Ct) values were normalized to Uni 6 Spike-in within the same cDNA concentration. **f** Identification of target genes of miR-1. TargetScan and miRDB were used to screen for miR-1 target genes in the dog database. A Venn diagram indicated that the TargetScan and miRDB datasets shared 293 genes. **g**, **h** KEGG and Reactome analyses of the top 100 genes identified via both TargetScan and miRDB. **i**, **j** Changes in the expression of potential target genes of miR-1 at the mRNA and protein levels. HACE1, C5orf51, PTPLAD1, GLCCI1, and MMD were decreased when pTDEs were administered. Ct values were used to normalize the levels of target genes to those of A5B, and the starting cDNA concentration was the same for all samples. The protein levels of HACE1 and the HACE1 target gene Rac1 were analyzed via Western blotting. The statistical analysis is presented. The error bars represent the means ± SEMs. Two-way ANOVA was used to compare groups. *P < 0.05, ***P < 0.001.

We identified 307 and 249 miRNAs from pTDEs and mTDEs, respectively (Fig. 4a, b and Supplementary Table 2, 3). The term “miRNA involved in cell proliferation” was the top Gene Ontology (GO) term for the pTDE miRNAs (Supplementary Fig. 6f, top). The term ‘regulation of stem cells’ was the main enriched term for mTDE miRNAs (Supplementary Fig. 6f, bottom). Since one miRNA silences multiple genes, we investigated miRNA target genes using TargetScan and miRDB, which include reference genomes for dogs (Supplementary Table 4). Kyoto Encyclopedia of Genes and Genomes (KEGG) pathway analysis revealed that the target genes of the pTDE miRNAs were related to cellular proliferation pathways, such as the cAMP, cGMP-PKG, PI3K-Akt and TNF signaling pathways (Supplementary Fig. 6g). Based on the GO and KEGG analyses, exosomal miRNAs might be the main factor mediating the inhibition of proliferation of metastases induced by pTDEs. Next, we identified the miRNAs that were differentially expressed in pTDEs and mTDEs. Notably, among the miRNAs found in the pTDEs, cfa-miR-1-1 and -2 had the highest expression levels (Fig. 4c). In addition, from the miRNA–gene network analysis, miR-1 had the highest number of nodes among the miRNAs and the highest degree of miRNAs known to play a distinct role in tumor suppression (Fig. 4d).

Interestingly, the difference in the expression pattern of miR-1 between RNase-treated pTDEs and mTDEs corresponded with the difference between CHMp and CHMm cells (Fig. 4e). We integrated two databases (TargetScan and miRDB) that can retrieve miR-1 target genes and sorted 293 common genes (Fig. 4f). KEGG and Reactome analyses of the miR-1 target genes revealed a significant association with the cell cycle (Fig. 4g, h). Next, we selected ten predicted miR-1 target genes (HACE1, C5orf51, PTPLAD1, GLCCI1, MMD, GJA1, THSB4X, SCAF11, WNK3, and SMIM14) and assessed whether they were affected by pTDEs via qRT‒PCR. The levels of HACE1, C5orf51, PTPLAD1, GLCCI1, and MMD were significantly decreased by pTDE treatment in CHMm cells (Fig. 4i). In contrast, the other five genes did not significantly change (Supplementary Fig. 7a). Treatment with pTDEs also reduced the protein level of HACE1, which subsequently resulted in an increase in the level of the Rac1 protein, a target of the HACE1 E3 ligase^33,34^ (Fig. 4j). The regulation of the miR-1-HACE1-Rac1 axis could help explain how pTDEs cause growth inhibition in recipient metastatic cells. Taken together, these findings indicate that miR-1 in pTDEs can reduce HACE1 levels in recipient cells and lead to the accumulation of Rac1, which increases ROS levels and subsequently induces cell cycle arrest after DNA damage, slowing cell proliferation. Overall, pTDEs from primary tumors express miR-1, which might have a role in the suppression of metastasis.

### pTDEs-miR-1 have an antimetastatic effect in an in vivo mouse model

To verify that the inhibition of metastatic growth by pTDEs is due to high levels of miR-1, we treated CHMm cells with miR-1 (Supplementary Fig. 8). Treatment with liposomes containing miR-1 dramatically decreased the expression of the six target genes of miR-1 (Supplementary Fig. 8a). Cell viability and growth were also significantly reduced in the group treated with miR-1 (Supplementary Fig. 8b, c). Additionally, similar to conventional pTDE treatment, miR-1 treatment increased the proportion of cells in the G2/M stage of the cell cycle (Supplementary Fig. 8d). Furthermore, similar to the effects of pTDEs, miR-1 increased intracellular ROS levels and genomic instability (Supplementary Fig. 8e, f). Finally, miR-1 increased the levels of γ-H2A and Rac1 and decreased the level of HACE1 (Supplementary Fig. 8g). Overall, similar to the effects of pTDEs, miR-1 induced genomic instability, promoted G2/M phase retention, and suppressed proliferation.

**Fig. 5:**
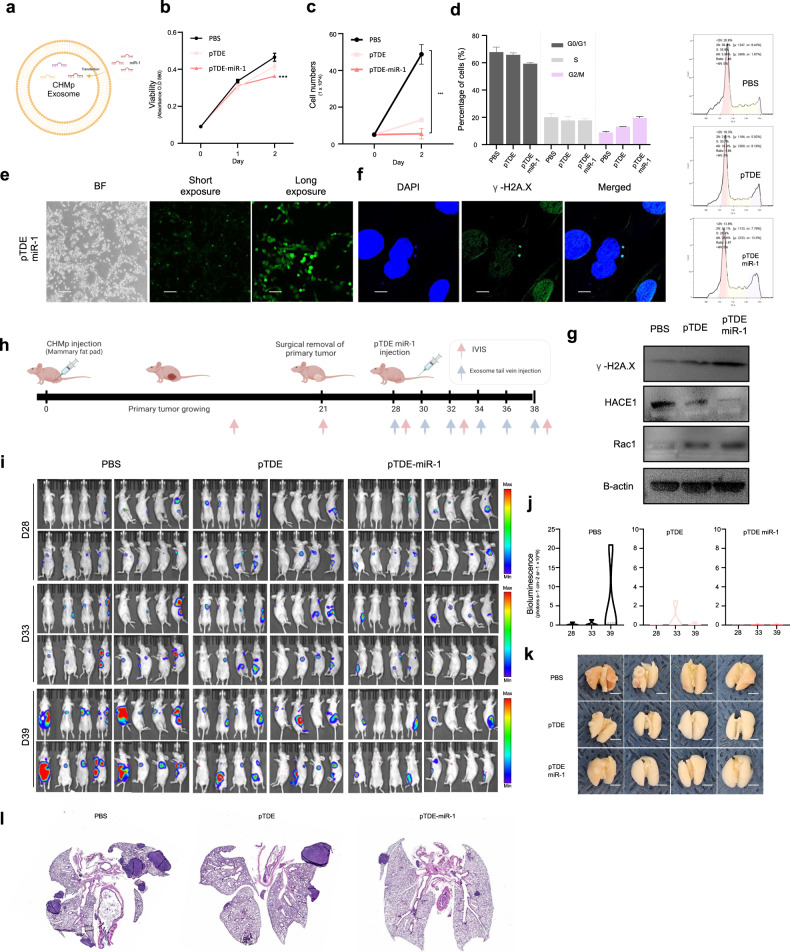
pTDEs-miR-1 had therapeutic effects in a mouse model. **a** Schematic illustration of miR-1 transfection into pTDEs. **b**–**d** Viability, proliferation and cell cycle assays were conducted in CHMm cells treated with PBS, pTDEs, or pTDEs-miR-1. Viability and proliferation were further decreased in cells treated with pTDEs-miR-1 compared with those treated with pTDEs. **d** Compared with those in the pTDE treatment group, the number of cells in the G2/M phase was further increased in the pTDE-miR-1 treatment group. (Left) Quantification of cell cycle progression in CHMm cells treated with PBS, pTDEs, or pTDEs-miR-1. (Right) Representative flow cytometry plot for each treatment group. **e** Representative images showing cellular ROS levels; brighter green fluorescence indicates high ROS. pTDE-miR-1-treated cells produced more ROS than did pTDE-treated cells. Scale bar, 170 μm. **f** For measuring damaged DNA, γ-H2A.X (green) and DAPI (blue) stains were used. The white arrow indicates damaged DNA, which colocalized with γ-H2A.X and DAPI. Scale bar, 10 μm. **g** Western blot for H2A.X and miR-1 target genes. Downregulation of HACE1 and upregulation of Rac1 were shown by Western blot analysis. **h** Schematic illustration of the established experimental mouse model and injection schedule. The mice were inoculated with CHMp cells into the mammary fat pad to produce a primary tumor. On Day 21, the primary tumors were surgically removed. Seven days after surgery, the mice were treated with PBS (*n* = 4), pTDEs (*n* = 4) or pTDEs-miR-1 (*n* = 4). **i** Bioluminescence images of lung metastases from mice after treatment with PBS, pTDEs, or pTDEs-miR-1 six times at two-day intervals. Bioluminescence images were obtained once a week. **j** Graph showing the quantification of lung metastases in mice treated with PBS, pTDEs, or pTDEs-miR-1. **k** Images of mouse lungs after PBS and pTDE treatment. Top: PBS-treated group; middle: pTDE-treated group; bottom: pTDE-miR-1-treated group. **l** Representative H&E staining of lung tissues from the metastasis model. Left two, PBS-treated group; middle two, pTDE-treated group; and right two, pTDE-miR-1-treated group (4X). The statistical analysis is presented. The error bars represent the means ± SEMs. Two-way ANOVA was used to compare groups. *P < 0.05, **P < 0.01, and ***P < 0.001. The results are presented as the mean ± SD (4 mice were used for each group). Figure 5a was created with BioRender.com.

We then engineered pTDEs overloaded with miR-1 (pTDEs-miR-1); this process resulted in an approximately 500-fold increase in the miR-1 concentration compared to that in pTDEs (Fig. 5a). Compared with those of pTDEs, the properties of pTDEs-miR-1 were significantly enhanced; for example, target gene levels, cell viability, and cell growth were further decreased, and G2/M phase arrest, ROS accumulation, DNA damage, and dormancy were further promoted in the pTDE-miR-1-treated group compared to the pTDE-treated group (Fig. 5b–g and Supplementary Fig. 8h, i). These results demonstrated that primary tumor-mediated metastatic growth inhibition primary tumors by metastasis can be mediated by miR-1 present in pTDEs.

Next, we examined the ability of pTDEs-miR-1 to inhibit metastatic growth in an animal model. As shown in Fig. 1, after removal of the primary tumor from the CMT mouse model, mice confirmed to have metastases in a secondary organ (lung) were grouped and treated with PBS, pTDEs, or pTDEs-miR-1. The injection schedule is depicted in Fig. 5h. As indicated by the radiance data, metastatic growth was dramatically suppressed in the pTDE group but was mostly suppressed in the pTDEs-miR-1 group compared to that in the PBS group (Fig. 5i, j). Lungs from the PBS-, pTDE-, and pTDE-miR-1-injected mice showed significant differences in metastatic nodule counts (Fig. 5k). Metastases were easily found in mouse lung tissues from the PBS group but scarcely found in those from the pTDE group, and the metastatic tumors were smaller in the pTDE group and much smaller in the pTDEs-miR-1 group than in the PBS group (Fig. 5l). Overall, these data strongly suggest that EVs derived from primary tumors and loaded with miR-1 can inhibit the formation and growth of metastases that have already formed in lung tissues.

### Blood exosomal miR-1 levels can be a diagnostic marker for metastases in clinical specimens

**Fig. 6:**
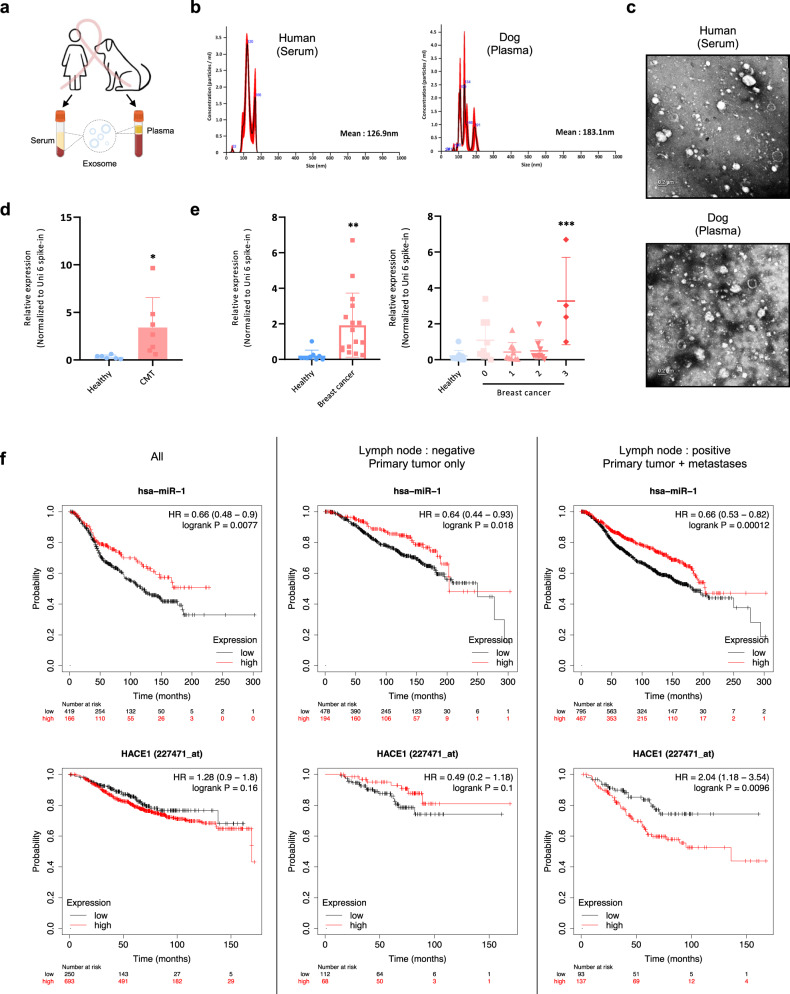
Exosomal miR-1 levels in biological fluids from dogs with mammary carcinoma and human breast cancer patients. **a** Schematic diagram of exosomes derived from human breast cancer patients and subjects with canine mammary carcinoma. **b** NTA was used to analyze the size of the exosomes. Size distribution of human serum-derived exosomes (left) and dog plasma-derived exosomes (right). **c** TEM image showing the external appearance of the exosomes. Top: human serum-derived exosomes. Bottom: dog plasma-derived exosomes. **d** The levels of miR-1 in the exosomes of dogs with canine mammary carcinoma (*n* = 7) and healthy controls (*n* = 6) were examined using qRT‒PCR and normalized to the Uni 6 spike-in as a control. **e** Expression levels of miR-1 in human breast cancer patients (*n* = 31) and healthy controls (*n* = 9) were examined using qRT‒PCR and normalized to the Uni 6 spike-in as the control. miR-1 was greater in breast cancer patients than in healthy controls, especially in late-stage (TNM stage III) patients compared to healthy controls. (stage 0: *n* = 9, stage I: *n* = 9, stage II: *n* = 9, stage III: *n* = 4) *P < 0.05, **P < 0.01, ***P < 0.001. **f** In silico Kaplan‒Meier analysis of breast cancer patients (http://kmplot.com/analysis/). OS curve comparing patients with high (red) and low (black) miRNA-1 expression. miR-1 (top) and HACE1 (bottom) expression in breast cancer patients with or without lymph node metastasis. Left: analysis of all patients. Middle: analysis of lymph node-negative patients. Right: analysis of lymph node-positive patients. Figure 6a was created with BioRender.com.

We then investigated whether exosomal miR-1 can be detected in the blood of individuals with CMT and HBC (Fig. 6a). Patient information is described in Supplementary Table 5. EVs were isolated from plasma and serum obtained from the patients and were found to be cup-shaped and less than 150 nm in size (Fig. 6b, c). We observed that the expression of exosomal miR-1 was greater in both CMT and HBC patients than in healthy controls; furthermore, the expression of exosomal miR-1 increased gradually with the progression of breast cancer (Fig. 6d, e). The largest amount of exosomal miR-1 was detected in the sera of Stage III patients, suggesting that primary tumors in locally advanced metastatic stages of breast cancer might generate an increased inhibitory signal from exosomal miR-1 to inhibit the growth of hidden metastases.

Next, we investigated the association between miR-1 expression and patient survival using Kaplan‒Meier plotter. We found that the high miR-1 expression group had better overall survival (OS) than the low miR-1 expression group for both lymph node-negative and lymph node-positive patients, as well as for all patients. High levels of miR-1 in tumor tissues may lead to high levels of exosomal miR-1 in the blood, which could result in further suppression of metastasis. However, the OS patterns depending on HACE1 expression differed between groups based on metastasis status, as high HACE1 expression was associated with worse OS only in the presence of metastasis (Fig. 6f). Overall, exosomal miR-1 is significantly increased in both human breast cancer and canine mammary tumors. Moreover, exosomal miR-1 levels increased with increasing tumor grade, indicating a potential association with the progression of metastasis.

## Discussion

One of the key questions regarding the mechanisms of metastasis is how the primary tumor controls the tumor cells that have disseminated from the primary site. Primary tumor-derived cells migrate to blood vessels to become circulating tumor cells (CTCs), invade blood vessels and enter several other organs. CTCs travel alone or in clusters and maintain a dormant state to evade immunosurveillance mediated by immune cells in the blood^35,36^. If these CTCs find a conducive environment, they exit circulation and establish a new site of residence. These disseminated cells settle and grow in preferred organs, a phenomenon known as organotropism. After arriving at a secondary site, DTCs emerge from dormancy and retain their stem cell properties to reinitiate tumor growth^37,38^. The emergence of clinically significant metastatic tumors is crucial because previously quiescent DTCs reactivate and regain stem cell-like properties that facilitate self-renewal and the potential for continued tumor growth^39^. However, the underlying mechanisms governing the transition between dormant and awakened states in these cells, including the factors that determine their ultimate fate at secondary sites, have not been fully elucidated. Recently, Borriello et al. showed that metastasizing tumor cells are kept in a dormant state at secondary sites in the lung by primary tumor-associated macrophages^4^. Primary tumors were reported to inhibit the growth of metastases by inducing apoptosis in DTCs directly or by regulating immune cells and endothelial cells in the tumor microenvironment^4,6,11,40–42^. Because primary tumors inhibit DTCs, when the primary tumor disappears, DTCs start to grow, regardless of where they are located, due to release from growth inhibition^43^. This finding is supported by the fact that early-stage surgical removal (on Day 4 after initial tumor implantation) of primary breast tumors in mice induces micrometastases in the lymph nodes because DTCs are present in these nodes at this stage^42^. Conversely, late-stage surgery (on Day 13) results in the formation of distant organ metastases, such as those in the lungs, as DTCs have sufficient time to disseminate and colonize these organs^44^. Therefore, surgical removal of primary tumors remains a controversial topic in the field of cancer research. While this treatment can improve patient survival and drug accessibility^45^, it can also promote the growth of metastases^46,47^. Here, we revealed that DTCs cannot grow in a dormant state because of the presence of EVs derived from primary tumors. We also showed that the absence of EVs due to the absence of a primary tumor allows the growth of DTCs, while primary tumor-derived EVs can inhibit the growth of DTCs, even exhibiting a therapeutic effect.

To demonstrate how primary tumors can inhibit the growth of metastases, we studied the fate of DTCs after the removal of primary tumors in an ideal model. The growth inhibition induced by pTDEs clearly suggested that the primary tumor induces antitumor effects to prevent the development of metastasis (Fig. 1). We demonstrated that this antitumor effect of EVs was caused by the accumulation of ROS and damaged DNA. However, DNA damage results in an increase in the percentage of cells in the G2/M phase of the cell cycle, leading to growth arrest. Next, we found that EVs in the tumor mass can exert effects similar to endostatin, which has been reported to be an antiangiogenic factor by Folkman^11^. Furthermore, pTDEs limit the metastatic outgrowth of DTCs by disrupting endothelial cell formation of capillary-like structures. Consistent with our findings, the clearance of tumor cells in the EV-injected mouse model was mediated by both slow-cycling tumor cells and antiangiogenic agents (Fig. 2). Other reports have indicated that primary tumors can induce antitumor immunity by priming T cells and macrophages^4,6^. Due to the immunodeficient nature of the mice used, we could not analyze the role of EVs in immunity. Further investigation is warranted to determine whether pTDEs behave similarly in immunocompetent mice.

To generalize the inhibitory effect of primary tumors, we examined the difference in stemness between primary tumors and metastases. Then, we confirmed the difference in miRNA composition within EVs between the two groups. EVs derived from *CD*44^*+*^*/CD*24^−^ populations of CIPp and CHMp cells have much greater antitumor activity than EVs derived from the whole CIPp cell population, suggesting that these *CD*44^+^/*CD*24^−^ populations are involved in the suppression of metastases (Fig. 3).

Analysis of exosomal miRNAs revealed that miRNA-1 is abundant in primary tumor-derived EVs and that overloading miR-1 in pTDEs significantly enhances their growth-inhibitory effect (Figs. 4–5). Consistent with previous reports that miR-1 inhibits tumor growth and metastasis^48^, miR-1 is a tumor-suppressive miRNA conserved in humans and dogs that inhibits growth and metastasis in patients with breast cancer^48^. Furthermore, our study showed that treatment with pTDEs resulted in decreased expression of HACE1, which is a target of miR-1. Decreased expression of HACE1 led to the accumulation of Rac1, which in turn elevated ROS levels and induced cell cycle arrest. These findings are consistent with previous studies showing that HACE1 controls ROS generation by catalyzing the ubiquitination of Rac1^33^. Taken together, these findings suggest that miR-1 is involved in the suppression of metastasis.

miR-1 expression in primary tumors is not confined solely to CMT, as evidenced by its detection in primary colorectal cancer samples obtained from human patients^49^. In human colorectal cancer cell lines, miR-1 expression was also greater in SW480 (primary tumor) cells than in SW620 (metastases) cells^50^. In addition, compared with SW620 cells, SW480 cells exhibit CD133^+^ stem-like characteristics^51^. Our findings in dogs provide a valuable basis for research in human patient-derived cell lines, given that miR-1 is conserved in both species. These consistent results between dogs and humans provide evidence of a relationship between primary tumors, CSCs, and miR-1. Notably, however, EVs contain not only miRNAs but also other nucleic acids, proteins, and lipids that might play roles in inhibiting metastases.

Next, we observed the intriguing potential of EVs to serve as biomarkers for both HBC and CMT owing to their ability to be detected in biological fluids. Notably, samples from subjects with HBC and CMT exhibited increased levels of exosomal miR-1, with a progressive increase observed in HBC patients with advancing TNM stage. We postulate that this difference may reflect the primary tumor’s augmented release of exosomal miR-1 upon DTC dissemination and colonization of distant organs. Furthermore, consistent with our clinical observations, we found that HACE1 gene expression was a predictor of inferior OS in patients with both primary tumors and metastases compared to patients with only primary tumors (Fig. 6).

Our study has several limitations. It is insufficient to make a generalize as we have only verified in canine mammary gland tumors without confirming it in various types of cancers. While we observed changes in the HACE1/Rac1 pathway upon miR-1 enrichment, we did not elucidate the direct molecular pathway responsible for the observed decrease in proliferation. Additionally, we did not clarify how the abundance of CSCs in primary tumors affects growth inhibition. In future studies, we plan to address these limitations by including other primary tumor and metastatic cell lines for comparison and conducting additional in-depth research to elucidate the role of exosomal miR-1. Nevertheless, our research has initiated a new era of exploration into the inhibitory effect of EVs originating from primary tumors on metastasis, laying the foundation for future studies in this field.

In summary, we have provided the first evidence that EVs secreted by CSCs in primary tumors can impede metastatic growth. Moreover, our investigations provide a molecular basis for the EV-mediated suppression of DTC proliferation. Specifically, pTDEs harboring miR-1 exert dual effects: they induce ROS-mediated genomic instability and cell cycle arrest in metastatic cells while also impeding angiogenesis in nearby endothelial cells. Furthermore, our findings highlight that the most crucial factor in inhibiting metastatic growth is miR-1, which, when overexpressed in pTDEs, has therapeutic potential. Additionally, the increase in exosomal miR-1 levels in the serum with the progression of breast cancer suggests its potential use as both a diagnostic marker and a therapeutic agent.

### Supplementary information

Supplementary Information

Supplementary Table 1

Supplementary Table 2

Supplementary Table 3

Supplementary Table 4

Supplementary Table 5

## Data Availability

All the raw and processed exosomal miRNA-seq data for the CHMp and CHMm cell lines and their biological replicates generated in this study have been deposited in the NCBI Gene Expression Omnibus (GEO) database under accession number GSE213969.

## Code availability

The main code scripts used for miRNA-seq processing and visualization are described in detail and available at GitHub: https://github.com/snu-cdrc/exosomal-miRNA.
