# Supplementary material for: Extracellular vesicle-mediated transfer of miRNA-1 from primary tumors represses the growth of distant metastases
Source: Exp Mol Med. 2024 Mar 27;56(3):734–46. doi: 10.1038/s12276-024-01181-7 (PMC10985072; doi:10.1038/s12276-024-01181-7)
Supplement: Supplementary file 1 — Supplementary Information [file 12276_2024_1181_MOESM1_ESM.pdf]

Supplementary Information

Supplementary methods

Supplementary Figure 1. Establishment of a metastasis mouse model
Supplementary Figure 2. Isolation and characterization of EVs
Supplementary Figure 3. EV injection to metastasis model
Supplementary Figure 4. pTDEs are not related to apoptosis or cell mobility
Supplementary Figure 5. Primary tumor cell possesses more cancer stemness than metastases
Supplementary Figure 6. Small RNA sequencing of primary and metastasis-derived EVs
Supplementary Figure 7. Expression profiles of miR-1 target genes
Supplementary Figure 8. miR-1 transfection into CHMm cells and pTDEs

Supplementary Table 1. Information of the PCR primers and antibodies used
Supplementary Table 2. Identified EV miRNA lists
Supplementary Table 3. Identified novel miRNA lists
Supplementary Table 4. miRNA-mRNA interaction gene lists
Supplementary Table 5. Dog MGT and human BC patient information

Supplementary methods

Flow cytometry

Flow cytometry was performed to determine cancer stem cell populations in six canine mammary carcinoma cell lines. FACS analysis was performed as previously reported in our laboratory [1]. Briefly, single cells were suspended and fixed in cell staining buffer (BioLegend). Cells were incubated with the manufacturer's recommended concentrations of CD44 (PE, Biolegend, 338807) and CD24 (APC, Biolegend, 311117) antibodies on ice for 20 min in the dark. Next, the cells were washed twice, and the cell pellet was resuspended in 0.3 ml of cell staining buffer. For cell cycle analysis, cells were resuspended and fixed with 70% EtOH. Before analysis, fixed cells were stained with a mixture of 50 μg/ml propidium iodide (Sigma–Aldrich, P4864), 0.1 mg/ml RNase A (Invitrogen, 12091021), and 0.05% Triton X 100 (Sigma–Aldrich, 93443) in PBS for 30 min at 37 °C. For apoptosis detection, the FITC Annexin V Apoptosis Detection kit with PI was used (Biolegend, 640932). Stained cells were protected from light and kept on ice before flow cytometric and fluorescence-activated cell sorting (FACS). Flow cytometry was performed on a BD Biosciences FACS Aria II installed at the National Center for Inter-University Research Facilities (NCIRF) at Seoul National University.

RNA isolation and qRT–PCR

RNA isolation and real-time quantitative RT–PCR were performed as previously described [2]. Briefly, total RNA was isolated from cells using TRIzol™ reagent (Invitrogen, 15596026) according to the manufacturer's instructions. For cDNA synthesis, 2 μg of RNA was carried out using 5X cDNA Synthesis Master Mix (CellSafe, CDS-200). Exosomal miRNA was isolated using the miRNeasy kit (Qiagen, 217084), and the miRCURY LNA RT kit (Qiagen, 339340) was used for cDNA synthesis according to the manufacturer's instructions. Exosomes were lysed by Qiazol, 10 ng of RNA was prepared for reverse transcription, and UniSp6 RNA spike-in was used as an internal control. Real-time PCR was carried out

using CFX Connect (Bio-Rad). We used the endogenous control A5B for cell-derived RNA and the UniSp6 spike-in control for miRNA. Primers and Tm for PCR are shown in Supplementary Table 1.

MTT assay

The MTT assay was performed as previously reported [3]. In brief, exosome-treated cells were washed with PBS and incubated with 5 mg/ml MTT (3-(4,5-dimethylthiazol-2-yl)-2,5-diphenyltetrazolium bromide) solution (Invitrogen, M6494). After 1 hr at 37 °C incubation, the medium was removed carefully and washed once with PBS. Formazan crystals were dissolved in 200 µl of DMSO. Then, 100 µl of medium from each well was measured for light absorbance at 570 nm using an absorbance microplate reader (BioTek).

ALDEFLUOR assay

The ALEFLUOR assay was performed to quantify ALDH activity in cells using an ALDEFLUOR kit (Stemcell Technologies, 01700) according to the manufacturer's instructions. The fluorescent substrate of ALDH, BODIPY-aminoacetaldehyde (BAAA), is converted into green fluorescence BODIPY-amino acetate (BAA) by ALDH, which is examined through a fluorescence microscope. CHMp and CHMm cells were incubated with 10 µl of activated ALDEFLUOR for 1 hr. The cells were washed with PBS and observed under a fluorescence microscope (Echo).

Mammosphere formation assay

CHMp and CHMm cells were seeded onto a 30 cm^2 petri dish for low attachment at 5,000 single cells. Mammosphere formation consisted of serum-free DMEM-F12 (HyClone, SH30261.01) supplemented with 1X B27 (Gibco, 17504044), 20 ng/ml recombinant epidermal growth factor (EGF; Invitrogen, PHG0311), 10 ng/ml recombinant basic fibroblast growth factor (bFGF; Peprotech, 100-18B) and 100 U/ml penicillin–streptomycin (Gibco, 15140-163). Cells were grown for seven days in a humidified incubator at 37 °C with

5% CO_2. The mammospheres were evaluated by counting the number and size of spheres under bright field microscopy (Echo).

Transwell assay

Cell migration and invasion were analyzed using an 8.0-μm-pore polycarbonate membrane inserted in Transwell cell culture chambers (Corning, 3428) as previously reported by our laboratory [4]. Cells (0.5 x 10^5) and exosomes were seeded into the upper chamber in serum-free RPMI, and 750 μl of RPMI with 10% exosome-depleted FBS was added to the lower compartment. For the migration assay, the plate was incubated at 37 °C for 24 h. Unlike the migration assay, 100 μl of diluted Matrigel (1 mg/ml, Corning)-coated upper chamber was used and incubated at 37 °C for 48 hr for the invasion assay. After the incubation period, the insert was removed, and the cells were fixed with 4% PFA for 2 min. Next, fixed cells at the insert membrane were permeabilized by incubation with ice-cold methanol for 10 min. The insert was washed twice with PBS and stained with 5% crystal violet for 10 min at room temperature. Cells in the upper chamber of the insert were gently washed with PBS several times to remove excess dye. Cells on the upper surface of the microporous membrane were removed with a cotton swab, and pictures were taken under an optical microscope.

Tube formation assay

To determine whether exosomes inhibit angiogenesis, a tube formation assay was performed as previously reported [3]. Briefly, HUVECs were maintained with EBM ™ -2 Endothelial Cell Growth Basal Medium (Lonza, CC-3156) supplemented with 10% fetal bovine serum (FBS; Gibco, 1600044) and EGM™-2 Endothelial SingleQuots™ Kit (Lonza. CC-4176). HUVECs and exosomes were seeded together with EBM supplemented with 10% exosome-depleted FBS and EGM-SingleQuots for 24 h. After the incubation period, the cells were fixed with 4% paraformaldehyde and photographed under an optical microscope. The tube mesh size was measured using an ImageJ-angiogenesis analyzer and quantified from three microscopic

fields.

Intracellular ROS measurement

To measure cellular ROS levels, exosome-treated cells were washed with PBS and incubated with 100 μM 2',7'-dichlorodihydrofluorescein diacetate (H2DCFDA)-containing exosome-free medium for 1 hour at 37 °C. Cellular ROS converted H2DCFDA to green fluorescent 2',7'-dichlorofluorescein (DCF). After incubation, the medium was removed, cells were washed with PBS, and green fluorescence was observed under a fluorescence microscope.

Immunofluorescence staining and confocal analysis

Exosome-treated cells were fixed with 4% paraformaldehyde for 10 min at RT, washed with PBS twice, incubated with 0.1% Triton X-100 in PBS for 10 °C at RT for permeabilization and blocked with 5% BSA in PBS for 1 hr. Then, the cells were incubated with primary γ-H2A.X antibody (Abcam, ab11174) for 16 h at 4 °C, followed by incubation with fluorescently labeled secondary antibody (Abcam, ab150077). Fluorescence images were acquired using a confocal laser scanning microscope (Carl Zeiss) at NCIRF in Seoul National University.

Immunoblot analysis

Immunoblot analysis was performed as previously reported [5]. Briefly, cells and exosomes were lysed in RIPA buffer with a protease inhibitor cocktail. The primary antibodies used in this study were CD44 (Abcam, 1:2,500), CD24 (Abcam, 1:2,000), ALDH1A1 (Abcam, 1:2,500), β-Actin (1:5,000), TSG101 (Abcam, 1:2,000), Alix (Abcam 1:2,000), p38 & pp38 (Cell Signaling Technology, 1:2,000), p44/42&pp44/42 (Cell Signaling Technology, 1:2,000), HACE1 (Abcam, 1:2,000), Rac1 (Merck, 1:2,500) and γ -H2A.X (Abcam, 1:3,000) antibody. Except γ-H2A.X, phosphor-p38 and p44/42 antibodies, 5% skim milk was used for blocking. BSA (5%) was used for γ-H2A.X, phosphor-p38 and p44/42 antibodies for

blocking. Chemiluminescent signals in blots were visualized using an enhanced chemiluminescent system (BIOMAX, BWP0200). Detailed information on the antibodies used is shown in Supplementary Table 1.

References

1. Cho, H.-M. *et al.* Transplantation of hMSCs genome edited with LEF1 improves cardio-protective effects in myocardial infarction. *Molecular Therapy-Nucleic Acids* **19**, 1186-1197 (2020).
2. Lee, D., Lee, K.-H., Kim, D.W., Yoon, S. & Cho, J.-Y. CXCL5 inhibits excessive oxidative stress by regulating white adipocyte differentiation. *Redox Biology*, 102359 (2022).
3. Chang, H.-K. *et al.* Inducible HGF-secreting human umbilical cord blood-derived MSCs produced via TALEN-mediated genome editing promoted angiogenesis. *Molecular Therapy* **24**, 1644-1654 (2016).
4. Kim, D.W. & Cho, J.-Y. NQO1 is required for β-lapachone-mediated downregulation of breast-cancer stem-cell activity. *International Journal of Molecular Sciences* **19**, 3813 (2018).
5. Jeon, S.A., Kim, D.W. & Cho, J.Y. Neural precursor cell-expressed, developmentally down-regulated 4 (NEDD4) regulates hydrogen peroxide-induced cell proliferation and death through inhibition of Hippo signaling. *The FASEB Journal* **33**, 14772-14783 (2019).

Supplementary Figure

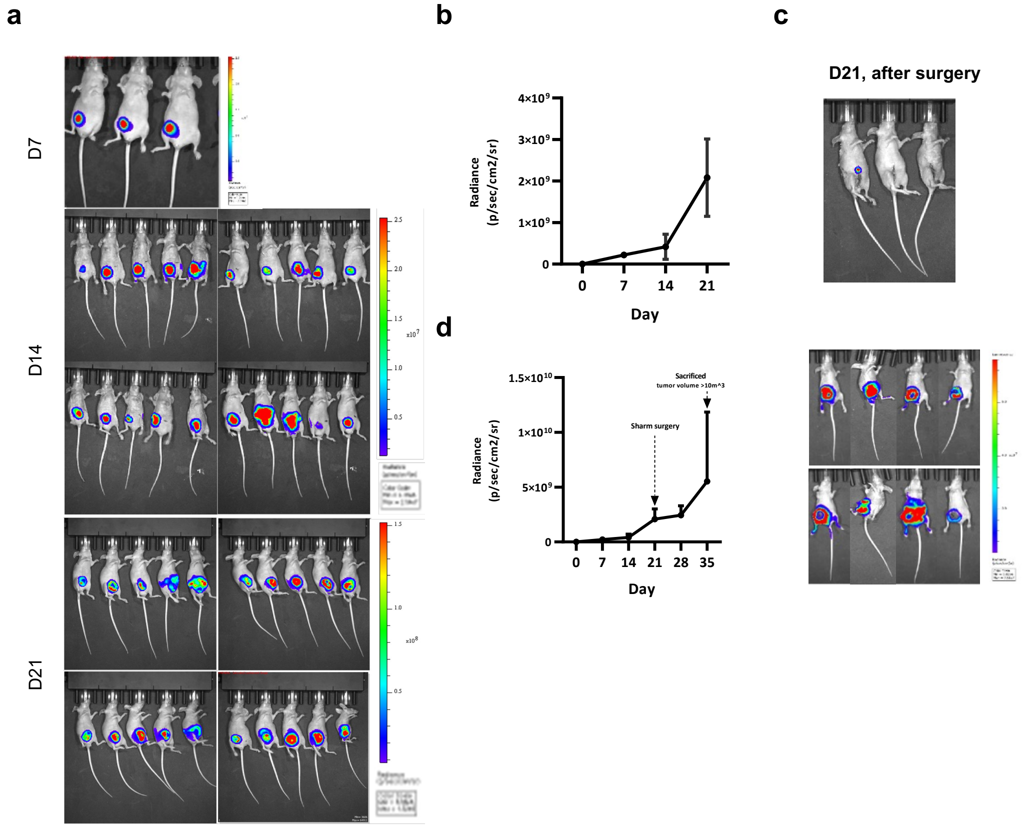

Supplementary Figure 1. Establishment of a metastasis mouse model. (a) Sequential bioluminescence images of the primary tumor growing nude mouse from day 14 to 21 using IVIS. **(b)** Bioluminescent intensity was analyzed using the IVIS imaging system. Over time, an increase in total flux intensity indicates growth of the primary tumor (imaged once a week, n=20). **(c)** Immediately after surgery, we confirmed that the primary tumors were not visible by IVIS, nor were metastases in secondary organs, such as the frequently metastasized lung. Representative bioluminescence images of primary tumor surgically resected mice. D21 bioluminescence images were acquired immediately after surgery. Left: primary tumor resident mouse, Middle & Right: primary tumor completely resected mouse. **(d)** Quantification of bioluminescence intensity and representative images of the sham group represent tumor growth (n=4). The dashed arrow indicates the day of surgery. Top : Day 28, Bottom : Day 35.

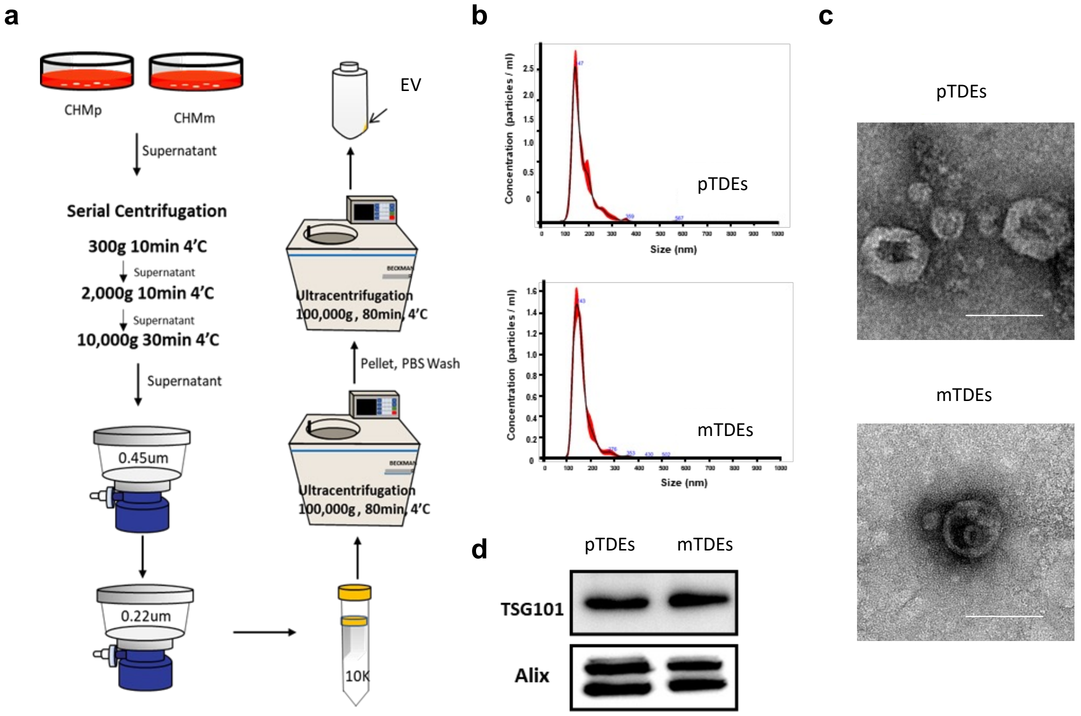

Supplementary Figure 2. Isolation and characterization of EVs. (a) Schematic illustration of EV isolation. Differential centrifugation, ultracentrifugation, and another ultracentrifugation were performed sequentially. **(b)** Nanoparticle tracking analysis (NTA) was used to analyze the size of EVs. Size distribution of pTDEs (top) and mTDEs (bottom). **(c)** Transmission electron microscopy (TEM) showed the external appearance of EVs. Top: pTDEs, Bottom: mDTEs. Scale bar = 100 nm.The white arrows indicate cup-shaped EVs. **(d)** EV markers TST101 and Alix were identified by Western blot.

a

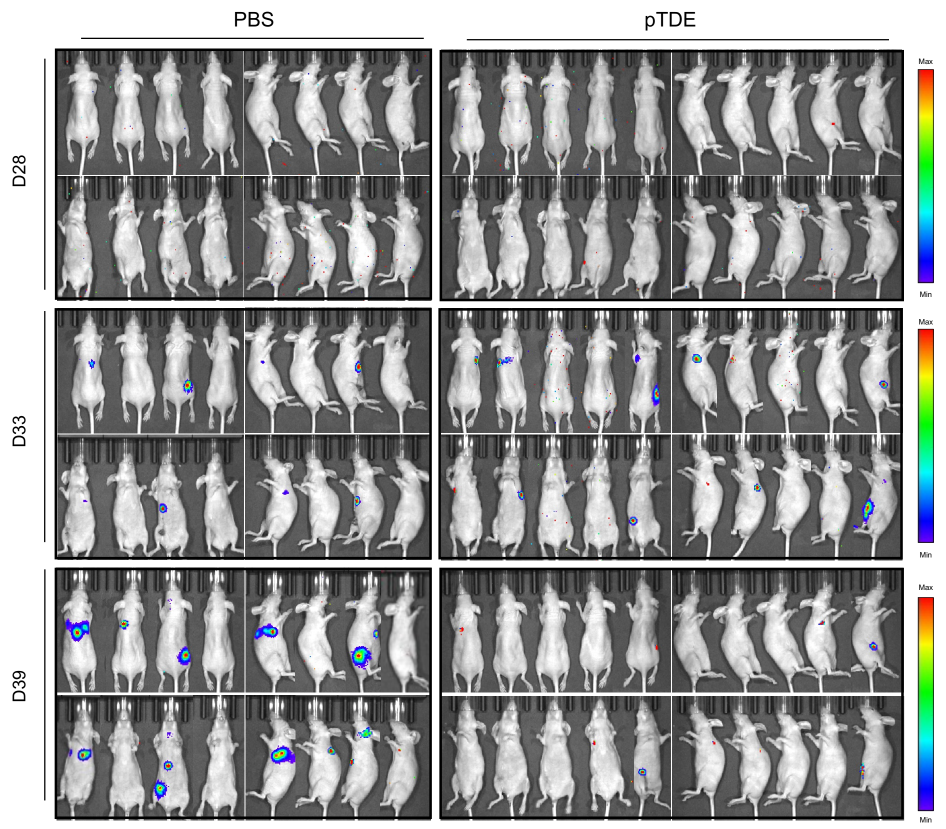

b

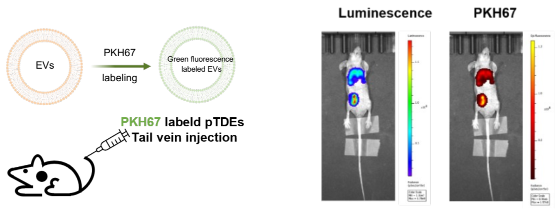

Supplementary Figure 3. EV injection to metastasis model. (a) Establishment of the PKH67-labeled EVs injection model. PKH67-labeled EVs were inoculated into mice via the tail vein and monitored by IVIS imaging. PKH67-labeled EVs accumulated at the primary site and metastases 48 h after injection. **(b)** Bioluminescence images of D28, D33 and D39. For each group of daily images, four mouse images were acquired at four positions (dorsal, ventral, right lateral and left lateral) to capture every possible signal from the mice.

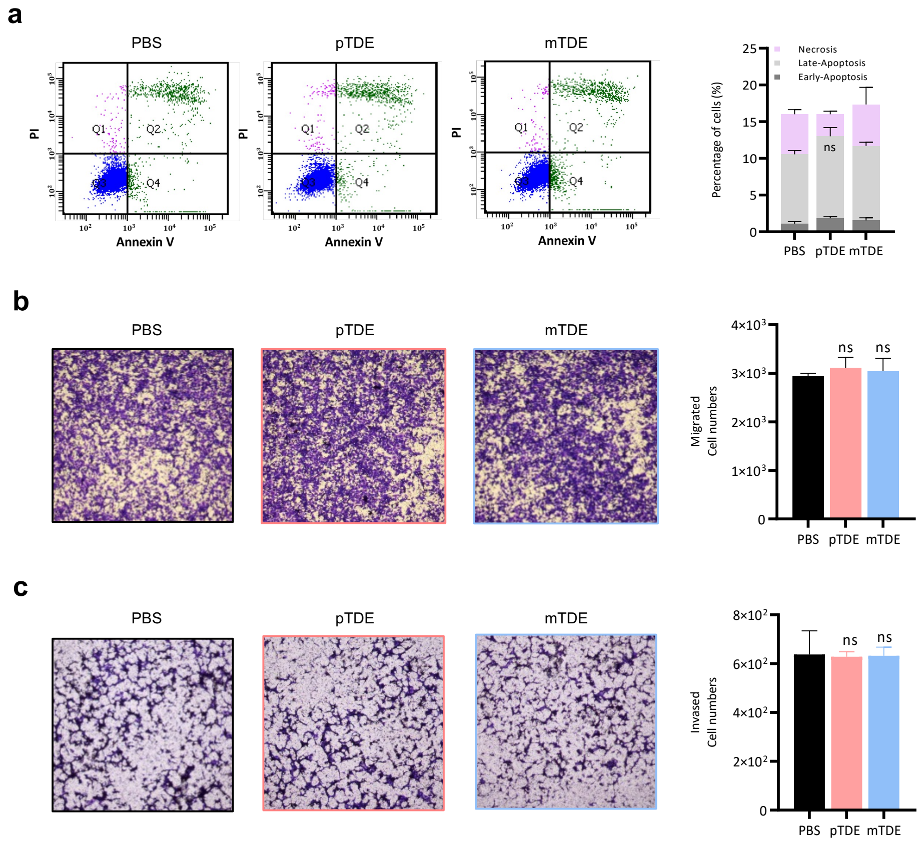

Supplementary Figure 4. pTDEs are not related to apoptosis or cell mobility. (a) EVs-treated cells were stained with Annexin V and PI to detect apoptosis. The upper left (necrosis), upper right (late apoptosis), and lower right (early apoptosis) were quantified by flow cytometry. **(b)** Trans-well migration assay with exosome-treated cells. Representative images of migrated cells were fixed and stained with crystal violet. The rate of migration was analyzed with ImageJ for quantification. There was no clear difference between groups. **(c)** Trans-well invasion assay in EVs-treated cells. Representative images of Matrigel-invaded cells were fixed and stained with crystal violet. The rate of invasion was analyzed with ImageJ for quantification. There was no clear difference between groups. All data are presented as the mean ± SEM (n=3). Two-way ANOVA and Tukey' HSD test was used to compare groups ; $^{**}P < 0.01$, *** $p < 0.001$, and ns, not significant. Results are presented as mean ± SD.

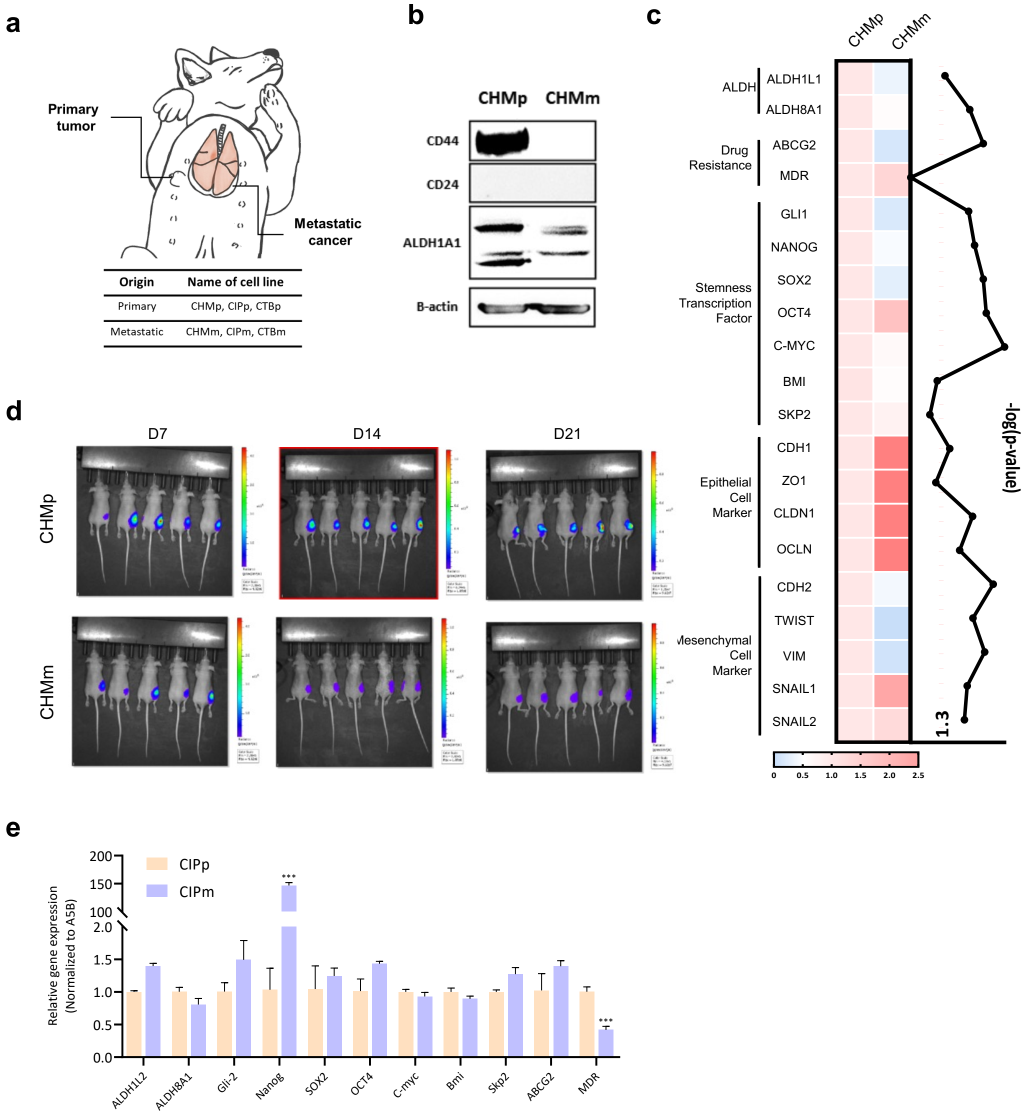

Supplementary Figure 5. Primary tumor cell possesses more cancer stemness than metastases. (a) Graphical illustration of the primary tumor and metastases of canine mammary carcinoma. **(b)** Western blot for CD44, CD24, ALDH1A1, and b-Actin. CD44 and ALDH1A1 were significantly increased in CHMp cells. b-Actin was used as an internal control. **(c)** Relative mRNA expression of ALDH, drug resistance, stemness transcription factor, epithelial cell and mesenchymal cell markers were quantified by qRT–PCR for CHMp and CHMm cells. The results were analyzed using the 2−ΔΔCT method using A5B as a reference gene. The color bar indicates fold changes compared to CHMp cells. Statistical significance between cells was assessed using Student's t test. The back line indicates –log(p value).

The red dotted line indicates $p < 0.005$. **(d)** Sequential bioluminescence images of the nude mice injected with CHMp and CHMm cells from day 7 to 21 using IVIS. **(e)** Gene expression of cancer stemness characteristics in CIPp and CIPm cells. ALDH (ALDH1L2 and ALDH8A1), stemness factors (Gli-2, Nanog, SOX2, OCT4, C-myc, and Bmi), and drug resistance (ABCG2 and MDR) genes were used for qRT–PCR. All data are presented as the mean Å} SEM (n=3). Student's t-test was used to analyze the data; The statistical analysis is presented. **$P < 0.01$ and ***$P < 0.001$.

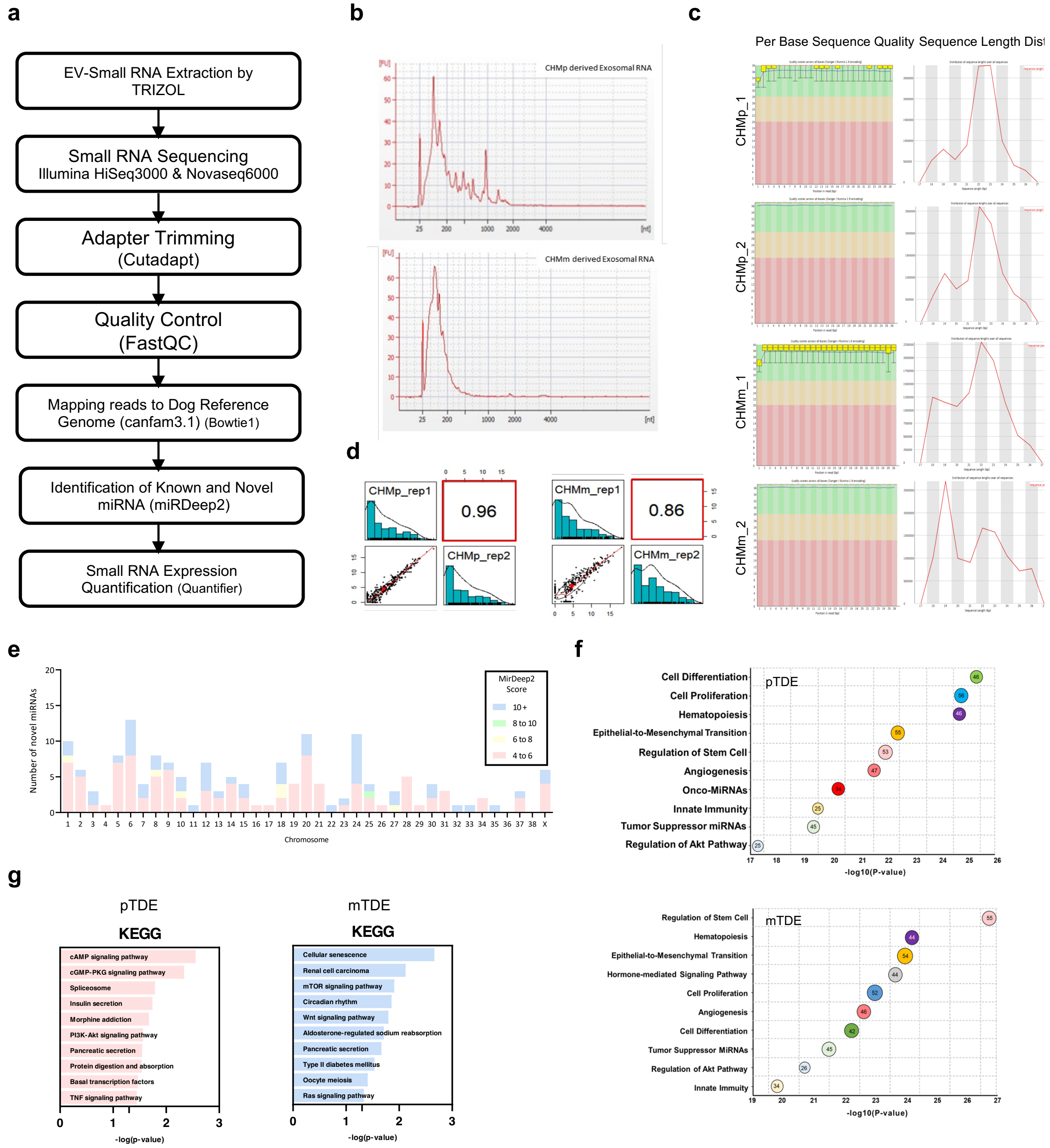

a
EV-Small RNA Extraction by TRIZOL
Small RNA Sequencing
Illumina HiSeq3000 & Novaseq6000
Adapter Trimming
(Cutadapt)
Quality Control
(FastQC)
Mapping reads to Dog Reference Genome (canfam3.1) (Bowtie1)
Identification of Known and Novel miRNA (miRDeep2)
Small RNA Expression Quantification (Quantifier)
b
CHMp derived Exosomal RNA
CHMm derived Exosomal RNA
[FU]
[nt]
c
Per Base Sequence Quality
Sequence Length Dist
CHMp_1
CHMp_2
CHMm_1
CHMm_2
d
CHMp_rep1
0.96
CHMp_rep2
CHMm_rep1
0.86
CHMm_rep2
e
MirDeep2 Score
10+
8 to 10
6 to 8
4 to 6
Number of novel miRNAs
Chromosome
f
pTDE
Cell Differentiation
Cell Proliferation
Hematopoiesis
Epithelial-to-Mesenchymal Transition
Regulation of Stem Cell
Angiogenesis
Onco-MiRNAs
Innate Immunity
Tumor Suppressor miRNAs
Regulation of Akt Pathway
-log10(P-value)
mTDE
Regulation of Stem Cell
Hematopoeisis
Epithelial-to-Mesenchymal Transition
Hormone-mediated Signaling Pathway
Cell Proliferation
Angiogenesis
Cell Differentiation
Tumor Suppressor MiRNAs
Regulation of Akt Pathway
Innate Immuity
-log10(P-value)
g
pTDE
KEGG
cAMP signaling pathway
cGMP-PKG signaling pathway
Spliceosome
Insulin secretion
Morphine addiction
PI3K-Akt signaling pathway
Pancreatic secretion
Protein digestion and absorption
Basal transcription factors
TNF signaling pathway
-log(p-value)
mTDE
KEGG
Cellular senescence
Renal cell carcinoma
mTOR signaling pathway
Circadian rhythm
Wnt signaling pathway
Aldosterone-regulated sodium reabsorption
Pancreatic secretion
Type II diabetes mellitus
Oocyte meiosis
Ras signaling pathway
-log(p-value)

Supplementary Figure 6. Small RNA sequencing of primary and metastasis-derived exosomes. (a) Flow chart of the exosomal miRNA sequencing procedure. **(b)** Bioanalyzer electropherograms of isolated CHMp (top) and CHMm (bottom) exosomal miRNA. **(c)** Per base sequence quality (left) and sequence length distribution (right) from the EV miRNA sequencing dataset. Top two; CHMp replicates (n=2), Bottom two; CHMm replicates (n=2). **(d)** Pearson correlation between EV miRNA sequencing replicates. Left: CHMp replicates (n=2), Right: CHMm replicates (n=2). **(e)** Novel miRNAs identified by the miRDeep2 score were distributed across the canine chromosomes. **(f-g)** Small RNA expression profiling of pTDEs and mTDEs. **(f)** Comparison of miRNA expression between pTDEs and mTDEs. **(g)** Differential expression of miRNAs between pTDEs and mTDEs was functionally enriched by the TAM2.0 tool. The size of the bubble indicates the corresponding involved gene number. KEGG analysis of pTDE miRNAs (top) and mTDE miRNAs (bottom).

a

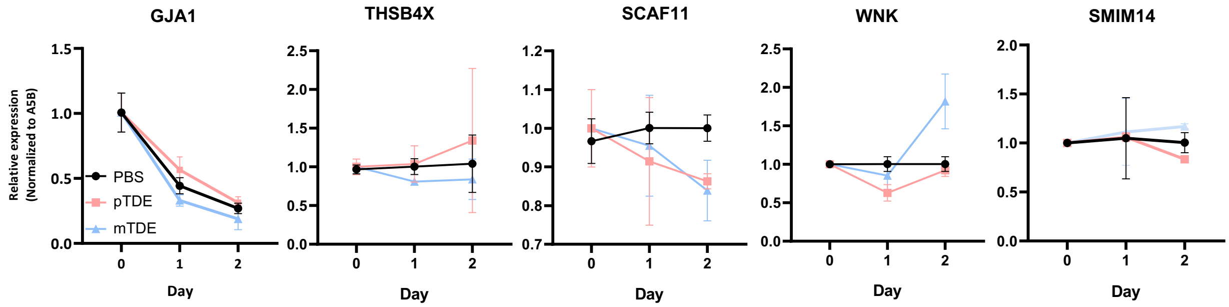

Supplementary Figure 7. Expression profiles of miR-1 target genes. (a) Endogenous mRNA expression of miR-1 target genes in CHMm cells with EVs treatment. All data are presented as the mean ± SEM (n=3). Unpaired Student's t test and two-way ANOVA were used to compare groups. **P < 0.01 and ***P < 0.001. ns not significant.

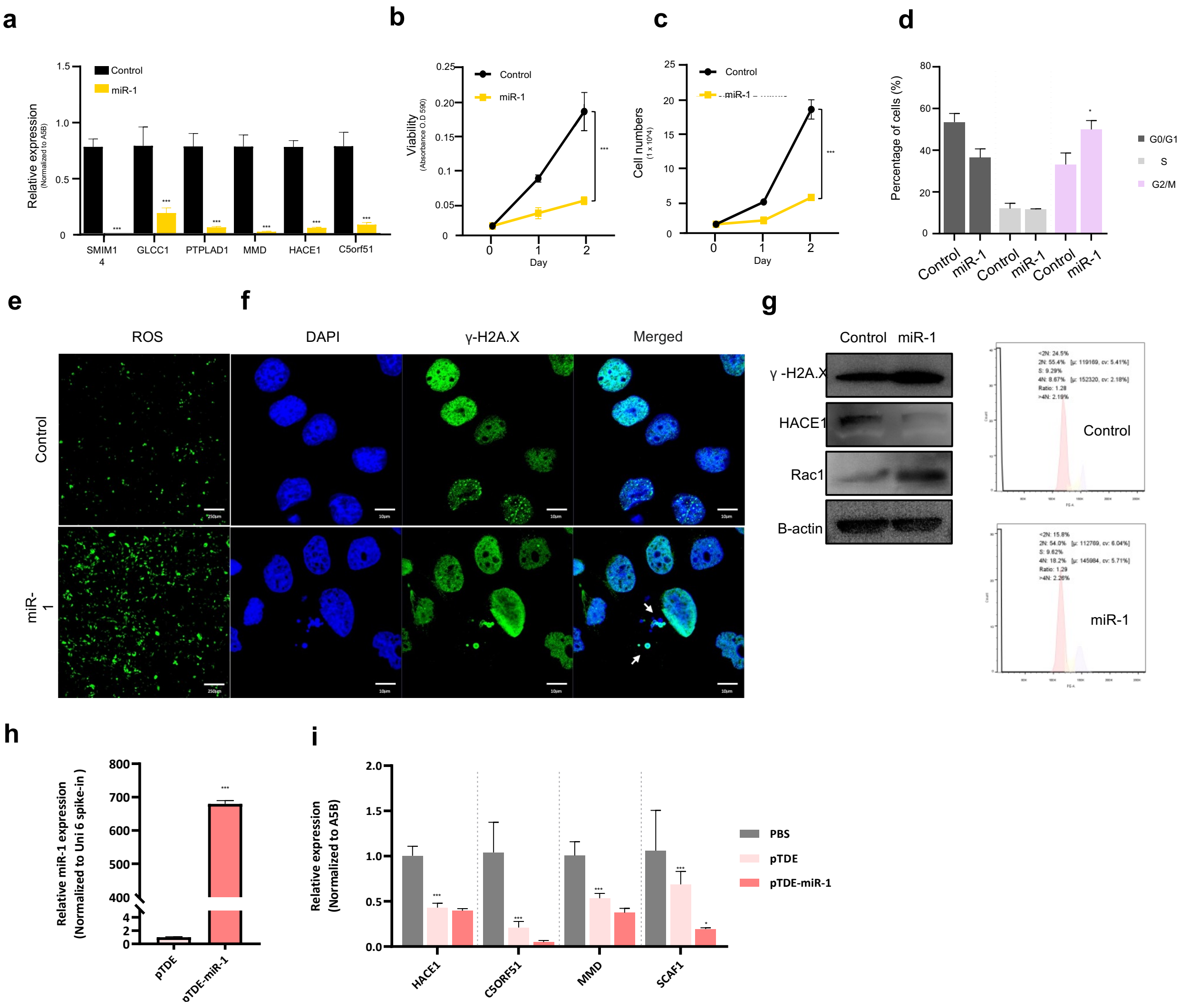

Supplementary Figure 8. miR-1 transfection into CHMm cells and pTDEs. (a-d) miR-1 overexpression in CHMm cells shows similar effects as pTDEs treatment. miR-1-transfected cells express lower levels of miR-1 target genes **(a)**, miR-1 decreased viability **(b)**, proliferation rate **(c)** and increased G2/M phase cell cycle arrest **(d)** in CHMm cells. (Top) Quantification of cell cycle states in CHMm cells treated with miR-1. (Bottom) Representative flow cytometry graph for each treated group. **(e)** Representative images showing cellular ROS levels. Brighter green fluorescence indicates high ROS. Scale bar, 250 μm. **(f)** For measuring damaged DNA, γ-H2A.X (green) and DAPI (blue) stains were used. The white arrow indicates damaged DNA, which colocalized with γ-H2A.X and DAPI. Scale bar, 10 μm. **(g)** Western blot for γ-H2A.X and miR-1 target genes. Downregulation of

HACE1 and upregulation of Rac1 were shown by Western blotting. **(h)** Increased miR-1 levels in pTDE-miR-1 compared to pTDEs. Uni 6 spike-in was used as the control. **(i)** Expression changes in the mRNA levels of potential miR-1 target genes. C5orf51, MMD and SCAF1 were decreased with pTDEmiR-1 treatment. Ct (cycle of threshold) values are normalized to A5B gene expression within the same cDNA concentration. Unpaired Student's t test was used to compare groups. (*$p < 0.05$, ***$p < 0.001$).
